# Bark and Grape Microbiome of *Vitis vinifera*: Influence of Geographic Patterns and Agronomic Management on Bacterial Diversity

**DOI:** 10.3389/fmicb.2018.03203

**Published:** 2019-01-08

**Authors:** Nicola Vitulo, Wilson José Fernandes Lemos, Matteo Calgaro, Marco Confalone, Giovanna E. Felis, Giacomo Zapparoli, Tiziana Nardi

**Affiliations:** ^1^Department of Biotechnology, University of Verona, Verona, Italy; ^2^Research Centre for Viticulture and Enology, Council for Agricultural Research and Economics-CREA, Conegliano, Italy

**Keywords:** *Vitis vinifera*, next-generation sequencing, 16S rRNA, grape microbiota, bark microbiota, wine, terroir

## Abstract

In recent years, the concept of “microbial terroir” has been introduced in the frame of the more renowned notion of “vitivinicultural terroir,’ since several studies demonstrated that wine characteristics are related to regional microbial community compositions. Most of the existing research focused on grape berries microbiota, since it can directly impact wine quality. In this work we studied, for the first time through next-generation sequencing, the epiphytic bacterial community of vine bark and its relationships with grape microbiota. The study was carried out in two Italian wine appellations (situated in different regions) to explore the impact of biogeography, and the influence of two agronomical practices (biodynamic and conventional) was evaluated as well. Overall, our results show that grapevine bark harbors a rich epiphytic microbiota and displays a higher microbial biodiversity than grape berry. Moreover, this study suggests that geographic and anthropogenic factors impact both bark and grape bacteriomes, but to a different extent. The evidence of a “microbial terroir” seems to be even more marked in bark than in berries, possibly due to its permanence over time and to its physical proximity with soil. The importance of vine trunk bark, as potential source of inoculum for grapes and as interesting bacterial diversity habitat, is evidenced. This opens new fields of investigation, not only for researchers that aim at describing this little-known habitat within the vineyard, but also for stakeholders from the wine industry that want to understand the roles of microorganisms on the entire winemaking process, from vineyard to cellar.

## Introduction

Vitivinicultural ‘terroir’ is defined as “a concept which refers to an area in which collective knowledge of the interactions between the identifiable physical and biological environment and applied vitivinicultural practices develops, providing distinctive characteristics for the products originating from this area. “Terroir” traditionally includes specific soil, topography, climate, landscape characteristics and biodiversity features” (International Organisation of Vine [OIV], 2010). In recent years, the concept of “microbial terroir” has been introduced, as the interactions between several microorganisms, from vineyard to cellar, have been shown to impact the final quality of wine (Bokulich et al., 2016; Liu Y. et al., 2017), and the biogeographical patterns thereof unveiled the microbial contribution to the terroir notion (Bokulich et al., 2014, 2016; Gilbert et al., 2014; Knight et al., 2015; Zarraonaindia and Gilbert, 2015; Belda et al., 2017; Miura et al., 2017; Mezzasalma et al., 2018; Morrison-Whittle and Goddard, 2018).

Most of the studies focused on the surface-microbiota of grape berries, which impacts on the sanitary state of grapes and can have a direct influence on the winemaking process and, therefore, on wine quality (Martins et al., 2012, 2014; Setati et al., 2012; Bokulich et al., 2016; Portillo et al., 2016; Salvetti et al., 2016; Grangeteau et al., 2017; Oliveira et al., 2018). At the same time, soil serves as a primary reservoir for potential vine-associated bacteria, and some epiphytic bacteria are common among aboveground plant parts and soil, suggesting that the physical proximity between soil and grapevine parts might facilitate microbial migration (Martins et al., 2013; Zarraonaindia et al., 2015; Mezzasalma et al., 2018). Among other vine parts that might share their microbiota with grape skin, such as roots, leaves and flowers, bark has received attention only in few research papers. In particular, Martins and co-workers showed that soil and bark host a greater diversity and species richness than grapes and leaves, and that bacterial populations revealed similarities between bark and soil (Martins et al., 2013). Morrison-Whittle and co-workers used next-generation sequencing (NGS) to examine the roles of soil, bark and fruit as source-habitats of the fungal diversity of ferments, showing that eukaryotic microbial populations increasingly resemble those present on vine bark as the fermentation proceeds (Morrison-Whittle et al., 2017; Morrison-Whittle and Goddard, 2018). Indeed, not only the micro-environments associated with soil and bark are generally considered nutrient-richer than leaves and undamaged grape (Martins et al., 2013), but also the trunk bark can be a stable habitat for microbes, being a permanent part of the vine, in contrast to ephemeral tissues as fruits and leaves. Nevertheless, despite its potential importance, the diversity of epiphytic microbiota on grapevine bark remains poorly described and, to date, we are aware of no characterization through NGS of its bacterial component.

This work aims to unveil the composition of bark bacterial communities and its relationships with grape berries microbiota, to study their biogeography across two Italian wine appellations, and also to investigate the impact of different agronomical practices on the composition of grape and bark bacteriomes. Bark-associated bacteria were monitored for the first time through 16S-NGS, during ripening season (at veraison and harvest time), and results were integrated with those obtained about grape-associated bacteria, sampled at harvest. The research was conducted on vineyards in Chianti DOCG and Monferrato DOC, two vocated viticultural areas of Italy situated in Tuscany and Piedmont, respectively, whose terroir has been object of previous studies due to the great interest of consumers in the arising wines (Amato and Valletta, 2017; Mocali et al., 2017). In each area, two vineyards were studied, differing for the agronomic practices employed (namely: biodynamic and conventional management).

## Materials and Methods

### Plant Materials, Study Sites and Sampling

Thirty-six samples of grape berries and trunk bark (inner rhytidome) were collected aseptically from *Vitis vinifera* vines in two different viticultural areas, Monferrato DOC (approximately 44°41′24.1^′′^N 8°37′48.4^′′^E), Piedmont, Italy and Chianti DOCG (approximately 43°40′47.1^′′^N 10°53′31.8^′′^E), Tuscany, Italy, in one vintage (2015), transported on ice, and stored at -80°C until processing. Local grape variety was Dolcetto for Monferrato DOC and Sangiovese for Chianti DOCG, according to respective product specifications (Consorzio, 2013; Regione Piemonte, 2017). For each area, two vineyards differing for agronomic management were studied (namely, biodynamic and conventional farming); details of management practices were gathered through interviews with agronomic consultants and vineyard managers (see “Acknowledgments” section and Supplementary Table S1). At each site, three sampling points were identified in distal spatial points of different rows, in which samples of grape berries and grape bark were collected and processed independently (representing three biological replicates). Grape samples were collected in September, few days before harvest; bark samples were collected in June (at veraison) and September (few days before harvest, together with grape berries). Therefore, twelve samples of grape berries represented two regions and two agronomic managements; 24 samples of grape bark represented two regions, two agronomic managements and two sampling times throughout the season.

### Microbial Cell Collection, Genomic DNA Extraction, and Sequencing

DNA Extraction from grape berries was carried out as described by (Salvetti et al., 2016) with some modifications. Initially, grapes were placed in a in a sterile 500 mL flask containing 100 mL solution of Phosphate-buffered saline (PBS) at pH 7.4, in order to wash them and release the all microorganisms from the surface. This step was processed twice consecutively at 23°C for 3 h with slow shaking. Through 0.45 μm Whatman nitrocellulose membrane filters (Sigma-Aldrich) the washing solutions were filtered and stored at 4°C until DNA extraction. DNA was extracted (one filter membrane independently) using the PowerWater R DNA Isolation Kit (MO BIO Laboratories Inc., Carlsbad, CA, United States), according to the instructions from Kit.

DNA Extraction from bark was carried out by placing 4 g of bark sample in a in a sterile 100 mL flask containing 20 mL solution of Phosphate-buffered saline (PBS) at pH 7.4, in order to wash them and release the all microorganisms from the surface. This step was processed at 23°C for 30′ with slow shaking. The washing solution was then centrifuged, and the pellet was stored at 4°C until DNA extraction. DNA was extracted using the PowerSoil DNA Isolation Kit (MO BIO Laboratories Inc., Carlsbad, CA, United States), according to the instructions from Kit for wet soil samples.

For each sample, total genomic DNA was quantified and checked for purity at A260/280 nm (Nanodrop, Thermo Fisher Scientific, United States). DNA sequencing was performed at BMR Genomics srl (Padua, Italy). Briefly V3–V4 regions of 16S rRNA genes were amplified using the primers Pro341F: 5′-CCTACGGGNBGCASCAG -3′ and Pro805R: Rev 5′-GACTACNVGGGTATCTAATCC -3′ (Takahashi et al., 2014). Primers were modified with forward overhang: 5′-TCGTCGGCAGCGTCAGATGTGTATAAGAGACAG -[locus-specific sequence]-3′ and with reverse overhang: 5′- GTCTCGTGGGCTCGGAGATGTGTATAAGAGACAG- [locus-specific sequence]-3′ necessary for dual index library preparation, following Illumina protocol^1^. Samples were normalized, pooled and run on Illumina MiSeq with 2 × 300 bp approach. At the end the sequence fastq files were demultiplexed.

### Bioinformatic Analysis

The fastq sequences were analyzed using DADA2 (Callahan et al., 2016) a new tool that implements an error correction model and allows to identify exact sample sequences that differ as little as a single nucleotide. The final output of DADA2 is an amplicon sequence variant (ASV) table which records the number of times each exact amplicon sequence variant was observed in each sample. DADA2 was run as described in https://benjjneb.github.io/dada2/tutorial.html using the default parameters. In order to improve the overall quality of the sequences, the reads were filtered and trimmed using the filterAndTrim function implemented in DADA2. To remove low quality bases at the end of the reads, the truncLen option was set to 280 and 220 for the forward and reverse fastq files, respectively. Moreover, to remove the adapter sequences at the 5′ end the trimLeft option was set to 17 and 21 (forward and reverse reads, respectively). The taxonomic assignment was performed using the naïve Bayesian classifier method implemented in DADA2 using as reference the GreenGene database.

A phylogenetic tree of the ASV was obtained using the function AlignSeq implemented in DEPHER (Wright, 2016) R package to create the multiple sequence alignment and the FastTree program (Price et al., 2010) to create the final tree.

### Statistical Data Analysis

Statistical analysis was performed on R (Version 3.4.4) using the following R packages: phyloseq (version 1.24.0) to facilitate the import, storage, analysis, and graphical display of microbiome census data (McMurdie and Holmes, 2013); Vegan (version 2.4.2) for PERMANOVA analysis (Oksanen et al., 2013, 2018); ALDEx2 (version 1.12.0) to inspect differential abundance between different conditions accounting for compositional nature of the data (Fernandes et al., 2013) as suggested by CoDaSeq package (version 0.99.1) (Gloor and Reid, 2016); mixOmics (6.3.1) for dimensional data reduction (Rohart et al., 2017; Cao et al., 2018). Data were pre-processed removing possible contaminants (mythocondrial and chloroplast sequences) and filtering too rare features (features with less than 10 read counts and present in less than 2 samples). PERMANOVA was computed with andonis2 function of Vegan package and betadisper function of the same package for graphical output. For alpha, beta diversity and PERMANOVA analysis, unweighted UniFrac metric distances were computed on log-transformed data adding a pseudocount value of 1 to avoid logarithm of 0. Differential abundance testing was performed by ALDEx2 package, as proposed by CoDaSeq package analysis pipeline, in different stratified subset of data in order to control for confounders variables. To have a qualitative information about most discriminant features in the dataset we compute sparse partial least squares discriminant analysis with plsda, tune.splsda and splsda functions of mixOmics R package. For the latter we follow default pipeline: normalizing data with total sum scaling normalization and adding a pseudocount value of 1 (to raw data) to avoid issues when computing centered log-ratios.

## Results

**FIGURE 1:**
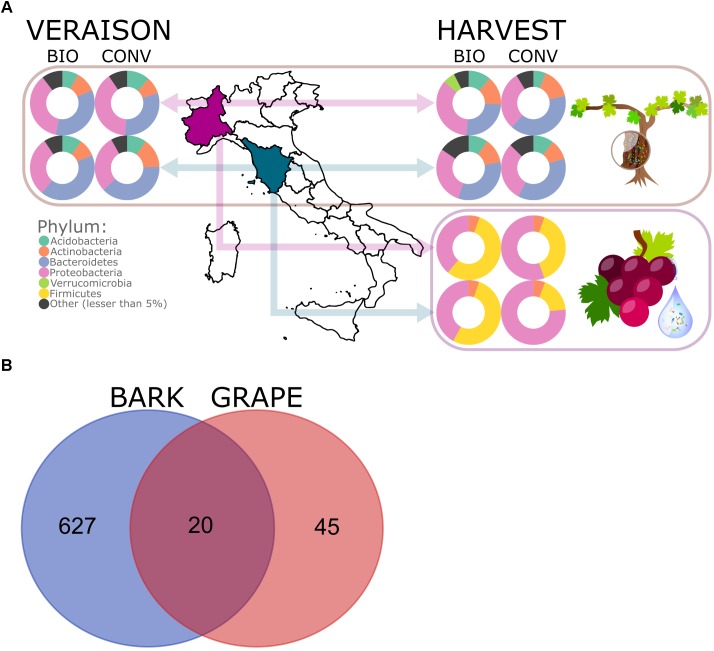
Experimental design and phylum-level abundance of bacterial epiphytes in bark and grape samples **(A)**. Venn diagram of shared bacterial ASVs between bark and grape **(B)**.

Three biological replicate samples of grape and bark were collected in four vineyards in two regions, where different agricultural management practices are employed (see Figure 1 for experimental design and Supplementary Table S1 for metadata). Surface bacterial community compositions were studied through high-throughput amplicon sequencing targeting the V3–V4 region of the 16S gene.

A total of 2.949.713 paired-end sequences (an average of 81.963 reads per samples) with a read length of 300 bp were obtained. After reads quality check, denoising and chimera filtering (see section “Materials and Methods” for details), 2.688 different Amplicon Sequence Variants (ASV) were obtained. The comparison of rarefaction curves (Supplementary Figure S1) as a function of sampling depth was performed and results showed that all curves are close to saturation, therefore the richness of the samples has been fully observed or sequenced (Rodriguez-R and Konstantinidis, 2014). Several filters based on taxonomic classification and ASVs abundance were applied in order to remove ASVs artifacts (see section “Materials and Methods” for more details).

A total of 692 different ASVs were obtained. The taxonomy classification allowed to identify 14 phyla, 36 classes (690 ASVs), 48 orders (663 ASVs), 70 families (608 ASVs), 67 genera (292 ASVs), and 15 species (38 ASVs).

### Dominating Bacterial Taxa, Diversity and Richness in Bacterial Communities

Proteobacteria and Actinobacteria were found ubiquitously across the whole experimental set (bark and grape samples in all vineyards, as shown in Figure 1A). The dominant bacterial phyla over bark samples were Acidobacteria, Actinobacteria, Bacteroidetes, Proteobacteria, Verrucomicrobia, and Chloroflexi; the dominant bacterial phyla across grape samples were Actinobacteria, Firmicutes, and Proteobacteria (Figure 1A). In general, bark presented more complex bacterial communities than grape surface, as can be observed also in the composition at class level (available in Supplementary Figure S2), with 35 and 5 entries, respectively. The Venn diagram reported in Figure 1B shows that 20 ASVs were shared between bark and grape, representing 30% of the 65 grape-associated ASVs and only 3% of the 647 bark-associated features.

To address the hypothesis that species richness and biodiversity vary with sample source (grape berries or bark), or with geographical and environmental variables such as region, season and agronomical practices, the intra group diversity estimation (alpha diversity), was calculated, using both the Shannon and Simpson’s diversity indexes.

**FIGURE 2:**
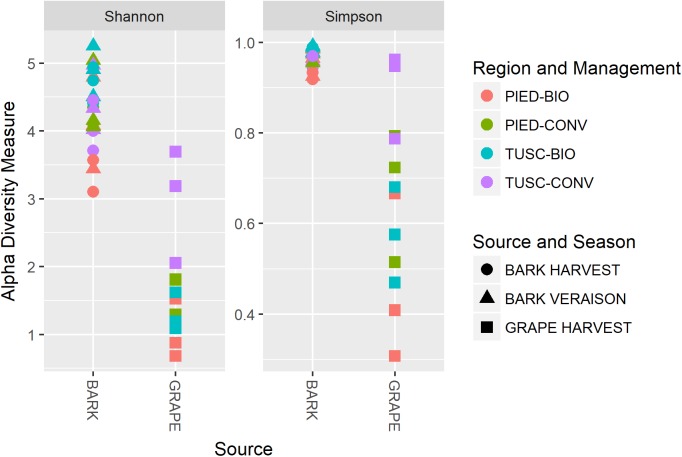
Alpha diversity calculated on the whole dataset, measured as Shannon and Simpson diversity indexes.

Results are plotted in Figure 2: both Shannon and Simpson’s indexes were significantly different between grape and bark samples (Wilcox test, *p* < 10^-6^). The latter sample showed higher biodiversity in both the studied regions, whereas no significant differences were found between the two regions nor between different agronomical practices or throughout the season.

### Diversity Analysis of the Epiphytic Bacterial Community on Bark and Grape

**FIGURE 3:**
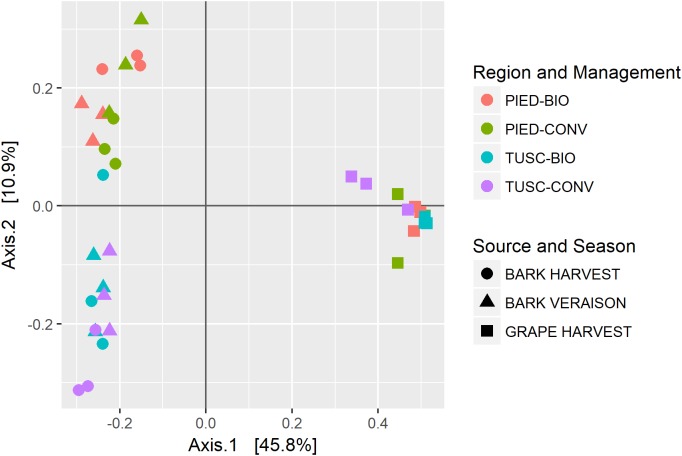
Beta diversity calculated on the whole dataset: PCoA using UniFrac distance.

In order to assess the amount of variation in species composition among the samples, we calculated the phylogenetic beta diversity based on unweighted UniFrac distance. The PCoA plot (Figure 3) shows a clear separation of the bacterial populations between bark and grape, visible on the axis 1 (explaining 45% of total variation). The second axis (accounting for 10% of variation) partially distinguishes the two regions among bark samples.

A Permutational Multivariate Analysis of Variance (PERMANOVA) was performed to explore the effects and significance of several variable such as sample Source (bark or grape), Region (Tuscany or Piedmont), agronomic Management (conventional or biodynamic) and Season (veraison or harvest) (Supplementary Table S2). The test revealed that all factors significantly affected microbial communities (Wilcox test, *p* < 0.05), and in particular that Source explained over 44% of the total variation (*p* = 0.0001) whereas Region and Management (2nd and 3rd most explanatory variables, respectively, *p* < 0.01 and *p* < 0.05) accounted together for less than 9%. Season (*p* < 0.05) and some interactions between factors that were also relevant, as Source:Region (*p* < 0.01), Source:Management, Month:Management and (*p* < 0.05), accounting for 2–4% of variance each.

As grape and bark bacterial communities clearly segregate, as testified by PCoA (Figure 3) and PERMANOVA results (Supplementary Table S2), we decided to perform further analyses on the two data-sets independently.

**FIGURE 4:**
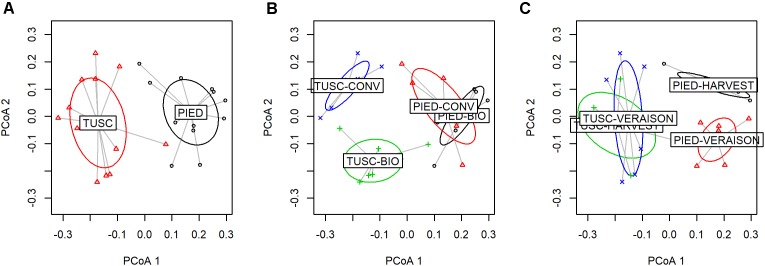
Factor analysis on bark dataset (PCoA based on UniFrac distances). Sample variable ordination by: Region **(A)**, Region and Management **(B)**, and Region and Season **(C)**.

At first, using PERMANOVA analysis we tested the effect of Region, agronomical Management and Season on the bark microbial community. The results (Supplementary Table S3 and Figure 4A) showed that Region was the principal factor impacting on microbial population diversity (18% of variation, *p* = 0.0001) and that Management and Season were also significant (*p* < 0.01, R2 0.8 and 0.7, respectively). Some interactions were also significant, mainly Season:Management (*p* < 0.01). To further evaluate if Management and Season factors had a different impact on beta diversity in the different regions, we repeated the PERMANOVA analysis stratifying the samples according to the Region. Results show that Management had a higher impact in Tuscany (Figure 4B), while Season had a higher impact in Piedmont (Figure 4C).

**FIGURE 5:**
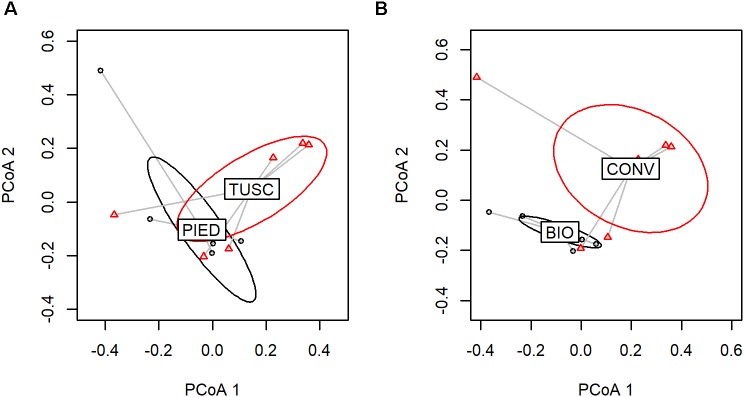
Factor analysis on grape dataset (PCoA based on UniFrac distances). Sample variable ordination by: Region **(A)** and Management **(B)**.

With regards to grape-associated bacterial community, the only factor that significantly affected its composition according to PERMANOVA was agronomic Management (*p* < 0.01), since Region impact was not statistically significant (Supplementary Table S4). Figure 5 shows groups-associated beta diversity, highlighting their separation.

### Drivers of Differentiation – Abundance Analysis and Discriminant Analysis

In order to identify taxonomic groups driving differences between bacterial communities, a differential abundance testing was performed on data stratified for sample source (see section “Materials and Methods” for details). Results for the bark data-set are reported in Supplementary Table S5. Six taxa were found differentially abundant between the two considered regions, five families were more abundant in Piedmont (namely: Acetobacteraceae, Caulobacteraceae, Hyphomicrobiaceae, Sporichthyaceae, Nocardioidaceae) and one in Tuscany (Chitinophagaceae). No differences were found considering the agronomic practices or sampling season and no features were found to be differentially abundant within the grape data-set.

**FIGURE 6:**
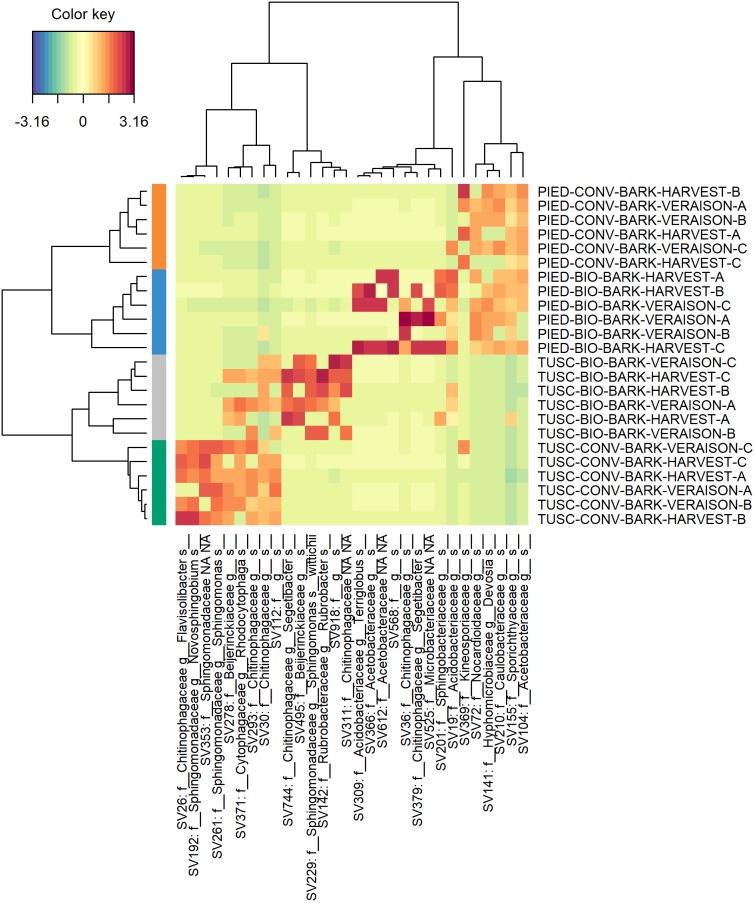
Heat map of discriminant features identified by the sPLS-DA analysis on bark dataset.

**FIGURE 7:**
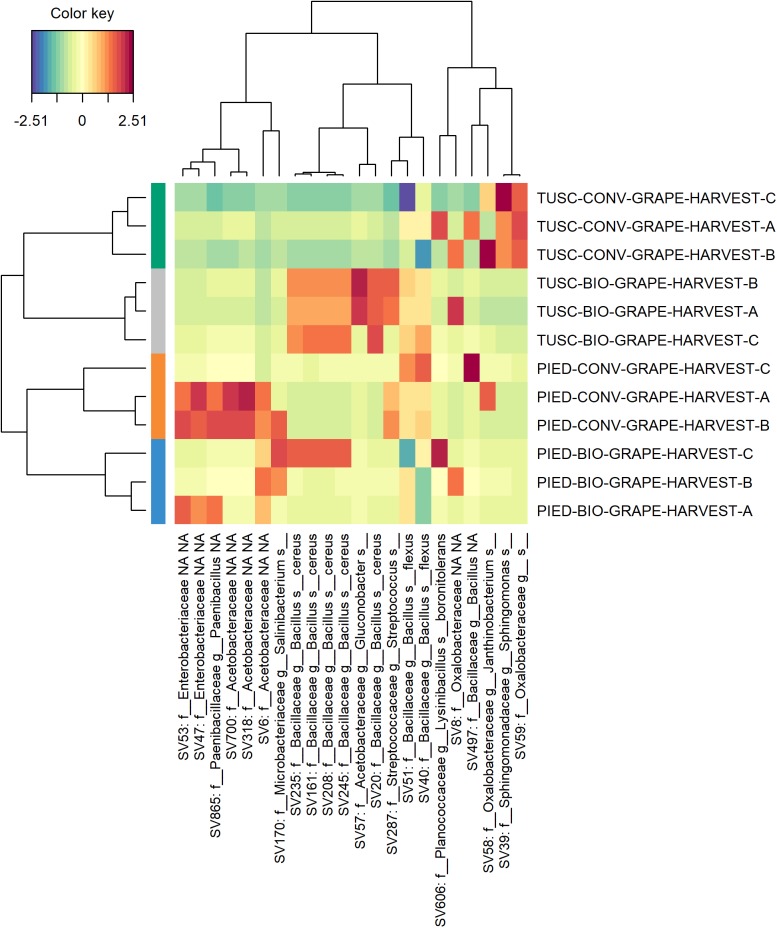
Heat map of discriminant features identified by the sPLS-DA analysis on grape dataset.

In order to better investigate the differences in the microbiota composition, a sparse Partial Least Squares Discriminant Analysis (sPLS-DA) was performed both on bark and grape data-sets independently, to select the most predictive and discriminative features. This analysis was carried out with the aim of clarifying more widely the influence of region and management on the bark and grape microbiome. Plots of sPLS-DA (components 1–2, 2–3) and of taxa contribution (loadings) on each component are available in Supplementary Material both for bark (Supplementary Figure S3) and for grape (Supplementary Figure S4) data-sets. The heatmaps reported in Figures 6, 7 resume the most discriminant taxa and their correlation with sample groups. With regards to bark data-set (Figure 6), it is clearly visible that samples from each vineyard group together, and that different features characterize each Region. Moreover, a partial overlap is visible between ASVs characterizing the biodynamic and the conventional vineyard in Tuscany, whereas in Piedmont the biodynamic-characterizing microbiota encompassed most of the features associated with the conventional vineyard. Also within the grape data-set (Figure 7), although much smaller, some features have shown to be suitable to differentiate the studied vineyards. Concerning the identity of taxa arisen from sPLS-DA, in addition to the families described in Supplementary Table S5 (significantly different in the abundance test in the bark data set), some more families were found to be important for characterizing bark samples (mainly: Sphingomonadaceae, Cytophagaceae, Rubrobacteraceae, Acidobacteriaceae), whereas some other families were the most informative in grape samples (mainly: Bacillaceae, Enterobacteriaceae, Paenibacillaceae, Oxalobacteraceae).

## Discussion

All the bacterial taxa identified in this work within the grape-associated microbiome were previously observed on grapes or in grape juices in other parts of the world (Zarraonaindia et al., 2015; Portillo et al., 2016; Miura et al., 2017; Morgan et al., 2017) and of the country (Marzano et al., 2016; Salvetti et al., 2016; Mezzasalma et al., 2017, 2018). All the phyla encircling relevant genera for winemaking (Stefanini and Cavalieri, 2018) were present, including Proteobacteria (encompassing spoilage and fermenting species) and Firmicutes [encompassing fermenting, innocent and spoilage species according to (Barata et al., 2012; Stefanini and Cavalieri, 2018)].

Concerning bark-associated bacteriome, this is the first 16S-amplicon based description, since previous studies either reported data on bacterial communities obtained with other techniques such as T_RFLP (Martins et al., 2013) or investigated fungal communities (Morrison-Whittle et al., 2017; Morrison-Whittle and Goddard, 2018). In general, our results are in line with previous studies, since grapevine trunk bark confirms to harbor significantly greater species richness than fruit, as previously observed both for bacteria (Martins et al., 2013) and for fungi (Morrison-Whittle et al., 2017). Moreover, the portion of overlap of bacterial community between the two habitats falls in line with similar findings on fungal communities, with 30% of grape-associated OTUs also present in bark samples (Morrison-Whittle et al., 2017). Furthermore, all the six bacterial classes previously detected on vine bark by culture-dependent methods (Martins et al., 2013) were found in our samples, within the 35 classes detected thanks to the NGS technique. This technique confirms to discern microbial taxa up to one order of magnitude more deeply than culture-based approaches in vineyard environment (Taylor et al., 2014). Finally, it is worth to note that all the main phyla characteristic of trunk bark (i.e., Actinobacteria, Bacteroidetes, Chloroflexi, and Verrucomicrobia) were previously found in vineyard soils (Zarraonaindia et al., 2015; Burns et al., 2016; Canfora et al., 2017; Novello et al., 2017) and are classified as having “absent/unknown effect” on wine fermentation (Stefanini and Cavalieri, 2018). This supports the finding of a previous work in which a comparison of bacterial genetic profiles from vineyards revealed similarities between bark and soil (Martins et al., 2013) and strengthens the interest of vine bark as an informative habitat for evaluating vineyard microbial biodiversity. Lastly, phyla detected ubiquitously among grape and bark samples (Acidobacteria, Proteobacteria) have been already described as widespread, being reported also in soils and leaves (Zarraonaindia et al., 2015; Liu S. et al., 2017).

Sample source (bark or grape) was found to be the major explanatory variable (44% explained) of microbial community structure of the studied vineyards, as previously observed also among soil, flower, leaf and grape samples (Martins et al., 2013; Zarraonaindia et al., 2015) and between grape and bark habitats for fungal species (Morrison-Whittle et al., 2017).

Analyzing separately bark and grape microbiomes, the first finding was that the Region of origin is the most important factor affecting bark bacterial populations (followed by agronomic Management and Season), whereas Management is the sole variable significantly affecting grape microbiome.

It is important to remark that, since the local grape varieties used in this study were different between Tuscany (Sangiovese) and Piedmont (Dolcetto) and grafted on different rootstocks, what we define “Region” is actually a variable encompassing confounding factors (cultivar and rootstock). Thus, we can observe a differentiation between bacteriomes that can be ascribed to different *terroirs* (in which different grapes are raised to give best performances for winemaking), rather than simply to different localities, as previously occurred also in other works (Pinto et al., 2015). Nevertheless, thanks to the results obtained in other studies (Bokulich et al., 2014; Portillo et al., 2016; Marasco et al., 2018; Mezzasalma et al., 2018), we can speculate that the contribution of biogeography to the observed differences is higher that the impact of grape variety and rootstock.

Besides, this work brings new comparative information about the microbial aspects of different regional terroirs in one of the most important wine producing countries [counting 74 DOCG and 333 DOC wine production appellations spanning across the nation (Federdoc, 2016)]. Indeed, although several recent works have addressed the study of grapevine related microbiota in Italy, (Campisano et al., 2014, 2017; Marzano et al., 2016; Salvetti et al., 2016; Stefanini et al., 2016; Canfora et al., 2017; Mezzasalma et al., 2017, 2018; Novello et al., 2017; Marasco et al., 2018), most of the studies focused on one single region (i.e., one or more viticultural sites within a wine production area), and none of the mentioned works included trunk bark in their examinations.

The finding that agronomic management affects more strongly fruit than bark microbiome has been previously proposed for fungi (Morrison-Whittle et al., 2017), nevertheless more studies on bark microbiome over years would be necessary to clarify the resilience of its microbiota and the impact of biogeography and agronomic practices on it.

Regarding the drivers of differentiation, statistical significance was found only for some bacterial taxa correlated with the Region of origin in the bark data-set. Among these, some bacterial families seem to be good candidates for containing markers of biogeography. Indeed, it is noteworthy that Acetobacteraceae have been previously shown to correlate to vitivinicultural regional microbial patterns and also to be important for predicting metabolite profiles of wines when found on grape berries or ferments (Bokulich et al., 2016). Moreover, Nocardioidaceae (here associated with Piedmont) were detected in bulk soil and rhizosphere in a recent NGS-based work carried out on vineyards in the same region and, specifically, in the same DOC area (Monferrato) analyzed in this study, although on another grape cultivar (Pinot Noir) (Novello et al., 2017). Both Caulobacteraceae and Hyphomicrobiaceae (here associated with Piedmont) families were recently identified among grapevine endophytes in northern Italy (Campisano et al., 2014, 2017), the former in roots and shoots, the latter in roots and stems.

Most of the above-mentioned features are part of the overlapping taxa between the microbiota characterizing biodynamic and conventional vineyards in Piedmont according to sPLS-DA (Figure 6). From the same analysis, some similarities between bacteria characterizing biodynamic vineyards in the two regions are also visible, since five on their 13 entries belong to the family of Chitinophagaceae (that is present only once in the conventional vineyards). Chitinophagaceae is a recently established family consisting of 13 genera (Kämpfer et al., 2011), of which many strains have been isolated or recovered in cow manure and compost (Storey et al., 2015; Ren et al., 2016), and that respond to the presence of cover crops (Liu S. et al., 2017). Both practices are used in the biodynamic farming (see Supplementary Table S1 for details about the vineyards object of this study), and have been recently reported to impact the vineyard soil microbiome composition (Chou et al., 2018; Hendgen et al., 2018). As some other authors recently suggested also for grape berries (Mezzasalma et al., 2017), biodynamic farming might influence the characteristic traits of resident microbiome. This explanation could also be inferred for our finding of *Bacillus cereus* in grape berries from biodynamic vineyards (mainly in Tuscany). Indeed, in the same paper (Mezzasalma et al., 2017), berries belonging to the biodynamic vineyards were described as rich in Bacillales typical of manure, including this species.

Overall, our results underline the importance of vine trunk bark, not only as a potential source of inoculum for grapes, but also as an interesting habitat to be characterized for monitoring microbial biodiversity in vineyards. Indeed, this niche is stable over seasons, relatively rich in nutrients, and harbors a rich epiphytic microbiota, thus its exploration allows to unveil much more biodiversity than the sole grape berry characterization. Moreover, our findings suggest that geographic and anthropogenic factors impact both bark and grape microbiome, but to a different extent. In particular, the evidence of a “microbial terroir,” already inferred by many authors for grape berries, seems to be even stronger in grapevine bark, possibly due to its permanence over time and to its physical proximity with soil. This opens new fields of investigation, not only for researchers that aim at describing this little-known habitat within the vineyard, but also for winemakers. Indeed, a new trend is imposing, in which the interest in metagenomic techniques is expanding from laboratories to the food industry, including wineries. The wine sector is nowadays gradually assimilating that microorganisms, including their ecological niches and population dynamics from vineyard to cellar, are crucial for the entire wine making process. Therefore, the knowledge of their role is emerging as a critical step for designing precision enology practices (Belda et al., 2017; Liu Y. et al., 2017).

In this context, further studies investigating fungal and bacterial communities of grapevine bark over years, performed in a larger number of grape varieties and viticultural areas, will allow to increase our understanding of the microbiome associated with bark, and to unveil its relations with grape berries microbiota. On the other hand, a predictive functional profiling of bark microbial communities from metagenomic 16S data, which would provide insights into the functional capabilities of the bark bacterial community, will be evaluated to complete and add biological significance to this study.

## Data Availability Statement

The datasets generated and analyzed during the current study are available in the NCBI SRA repository under the BioProject ID: PRJNA484333 (Biosamples accession numbers SAMN09763144-SAMN09763179).

## Author Contributions

TN set the project up and gathered the samples. NV, GF, GZ, and TN conceived and designed the experiments. WL and MCo performed the experiments. NV, WL and MCa analyzed the data. NV, MCa, and TN drafted the manuscript and figures. NV, GF, GZ, and TN critically revised and finalized the manuscript.

## Conflict of Interest Statement

The authors declare that the research was conducted in the absence of any commercial or financial relationships that could be construed as a potential conflict of interest.

## Footnotes

1 https://web.uri.edu/gsc/files/16s-metagenomic-library-prep-guide-15044223-b.pdf
