# Supplementary material for: Bark and Grape Microbiome of Vitis vinifera: Influence of Geographic Patterns and Agronomic Management on Bacterial Diversity
Source: Front Microbiol. 2019 Jan 8;9:3203. doi: 10.3389/fmicb.2018.03203 (PMC6331396; doi:10.3389/fmicb.2018.03203)

Supplementary Material

Bark and grape microbiome of *Vitis vinifera*: influence of geographic patterns and agronomic management on bacterial diversity

Nicola Vitulo[1], Wilson José Fernandes Lemos Junior[1], Matteo Calgaro[1], Marco Confalone[1], Giovanna E. Felis[1], Giacomo Zapparoli[1], Tiziana Nardi[2]*

[1] Department of Biotechnology, University of Verona, Verona, Italy

[2]Research Centre for Viticulture and Enology, Council for Agricultural Research and Economics (CREA), Conegliano, Italy

***Correspondence**:
Tiziana Nardi;
e-mail: tiziana.nardi@crea.gov.it,

Supplementary Table S1. Samples description and metadata

Source	Region and grape variety	Management	Season	Weed and cover crop management	Pest management - chemical	Pest management - Sulphur	Pest management - Coppper-sulphate
Bark	Piedmont, Dolcetto	Conventional	Veraison (June)	inter row:<br>no cover crop<br>under row:<br>chemical weeding	2 x Glyphosate (March)<br>2 x dithiocarbamate (30 days before veraison)<br>2 x systemic pesticides - (Dimethomorph, penconazole, 30 days before veraison)<br>1 x Metalaxyl (before veraison)	1 x 20 kg/ha<br>5 x 3 kg/ha	1 x Copper-sulphate<br>(< 3kg Cu2+/ha tot)
Bark	Piedmont, Dolcetto	Conventional	Harvest (September)				
Grape	Piedmont, Dolcetto	Conventional	Harvest (September)				
Bark	Piedmont, Dolcetto	Biodynamic	Veraison (June)	inter row:<br>permanent cover crop grassing-over<br>under row:<br>tillage	no	1 x 20 kg/ha<br>10 x 3 kg/ha	Copper-sulphate<br>(< 1,5kg Cu2+/ha tot)
Bark	Piedmont, Dolcetto	Biodynamic	Harvest (September)				
Grape	Piedmont, Dolcetto	Biodynamic	Harvest (September)				
Bark	Tuscany, Sangiovese	Conventional	Veraison (June)	inter row:<br>no cover crop<br>under row:<br>chemical weeding	1 x Glyphosate (March)<br>2 x dithiocarbamate (30 days before veraison)<br>2 x systemic pesticides - (Dimethomorph, penconazole, 30 days before veraison)<br>1 x Metalaxyl (before veraison)	1 x 20 kg/ha<br>5 x 3 kg/ha	1 x Copper-sulphate<br>(< 3kg Cu2+/ha tot)
Bark	Tuscany, Sangiovese	Conventional	Harvest (September)				
Grape	Tuscany, Sangiovese	Conventional	Harvest (September)				
Bark	Tuscany, Sangiovese	Biodynamic	Veraison (June)	inter row:<br>winter cover crop grassing-over<br>under row:<br>tillage	no	1 x 20 kg/ha<br>10 x 3 kg/ha	Copper-sulphate<br>(< 3kg Cu2+/ha tot)
Bark	Tuscany, Sangiovese	Biodynamic	Harvest (September)				
Grape	Tuscany, Sangiovese	Biodynamic	Harvest (September)				

Supplementary Table S2. PERMANOVA on whole dataset: Permutation test for Adonis under reduced model, Permutation: free, Number of permutations: 10000, Significance codes: 0 '***' 0.001 '**' 0.01 '*' 0.05 '.' 0.1 ' ' 1

	Df	SumOfSqs	R2	F	Pr(>F)	
Source	1	3,9677	0,4432	42,5904	1,00E-04	***
Season	1	0,2661	0,0297	2,8568	0,026797	*
Region	1	0,5229	0,0584	5,6127	0,0013	**
Management	1	0,3006	0,0336	3,2269	0,016598	*
Source:Region	1	0,3651	0,0408	3,9187	0,007699	**
Season:Region	1	0,2	0,0223	2,1471	0,067993	.
Source:Management	1	0,2391	0,0267	2,5664	0,035996	*
Season:Management	1	0,2631	0,0294	2,8239	0,027497	*
Region:Management	1	0,2129	0,0238	2,2858	0,058794	.
Source:Region:Management	1	0,1972	0,022	2,1164	0,070293	.
Season:Region:Management	1	0,1812	0,0202	1,9452	0,081792	.
Residual	24	2,2358	0,2498			
Total	35	8,9517	1			

Supplementary Table S3. PERMANOVA on bark dataset: Permutation test for Adonis under reduced model, Permutation: free, Number of permutations: 10000, Significance codes: 0 '***' 0.001 '**' 0.01 '*' 0.05 '.' 0.1 ' ' 1

	Df	SumOfSqs	R2	F	Pr(>F)	
Season	1	0,2684	0,0696	2,5596	0,0044	**
Region	1	0,7215	0,1872	6,8809	1,00E-04	***
Management	1	0,3155	0,0818	3,0086	0,0011	**
Season:Region	1	0,2003	0,0519	1,9099	0,027597	*
Season:Management	1	0,2633	0,0683	2,5108	0,005599	**
Region:Management	1	0,2256	0,0585	2,1511	0,012599	*
Season:Region:Management	1	0,1831	0,0475	1,7464	0,040596	*
Residual	16	1,6777	0,4352			
Total	23	3,8552	1			

Supplementary Table S4. PERMANOVA on grape dataset: Permutation test for Adonis under reduced model, Permutation: free, Number of permutations: 10000, Significance codes: 0 '***' 0.001 '**' 0.01 '*' 0.05 '.' 0.1 ' ' 1

	Df	SumOfSqs	R2	F	Pr(>F)	
Region	1	0,19581	0,1136	1,6891	0,15878	
Management	1	0,34695	0,2012	2,9928	0,0125	*
Region:Management	1	0,25426	0,1475	2,1933	0,07489	.
Residual	8	0,92741	0,5378			
Total	11	1,72443	1			

Supplementary Table S5. Differential Abundance Testing on bark dataset

	Order	Family	Genus	we.eBH	rab.win. PIED	rab.win. TUSC	diff.btw	diff.win	effect	overlap
SV104	o__Rhodospirillales	f__Acetobacteraceae	g__	0.0153806	83.232.281	-0.6673245	-8.482.918	4.282.898	-1.713.049	0.0794273
SV210	o__Caulobacterales	f__Caulobacteraceae	g__	0.0468839	72.472.368	-0.7901546	-7.371.347	4.889.769	-1.357.300	0.1222368
SV141	o__Rhizobiales	f__Hyphomicrobiaceae	g__Devosia	0.0365052	78.544.614	-0.7085851	-7.864.535	5.151.127	-1.338.663	0.1171876
SV155	o__Actinomycetales	f__Sporichthyaceae	g__	0.0060275	76.668.002	-0.4082511	-7.929.791	3.270.738	-2.351.805	0.0208342
SV72	o__Actinomycetales	f__Nocardioidaceae	g__	0.0373300	88.426.961	-0.6628193	-8.927.515	5.281.569	-1.383.913	0.1157349
SV30	o__[Saprospirales]	f__Chitinophagaceae	g__	0.0228141	-0.3729684	100.121.941	9.854.812	5.746.238	1.439.101	0.0884267

Supplementary Figure S1. Rarefaction curves

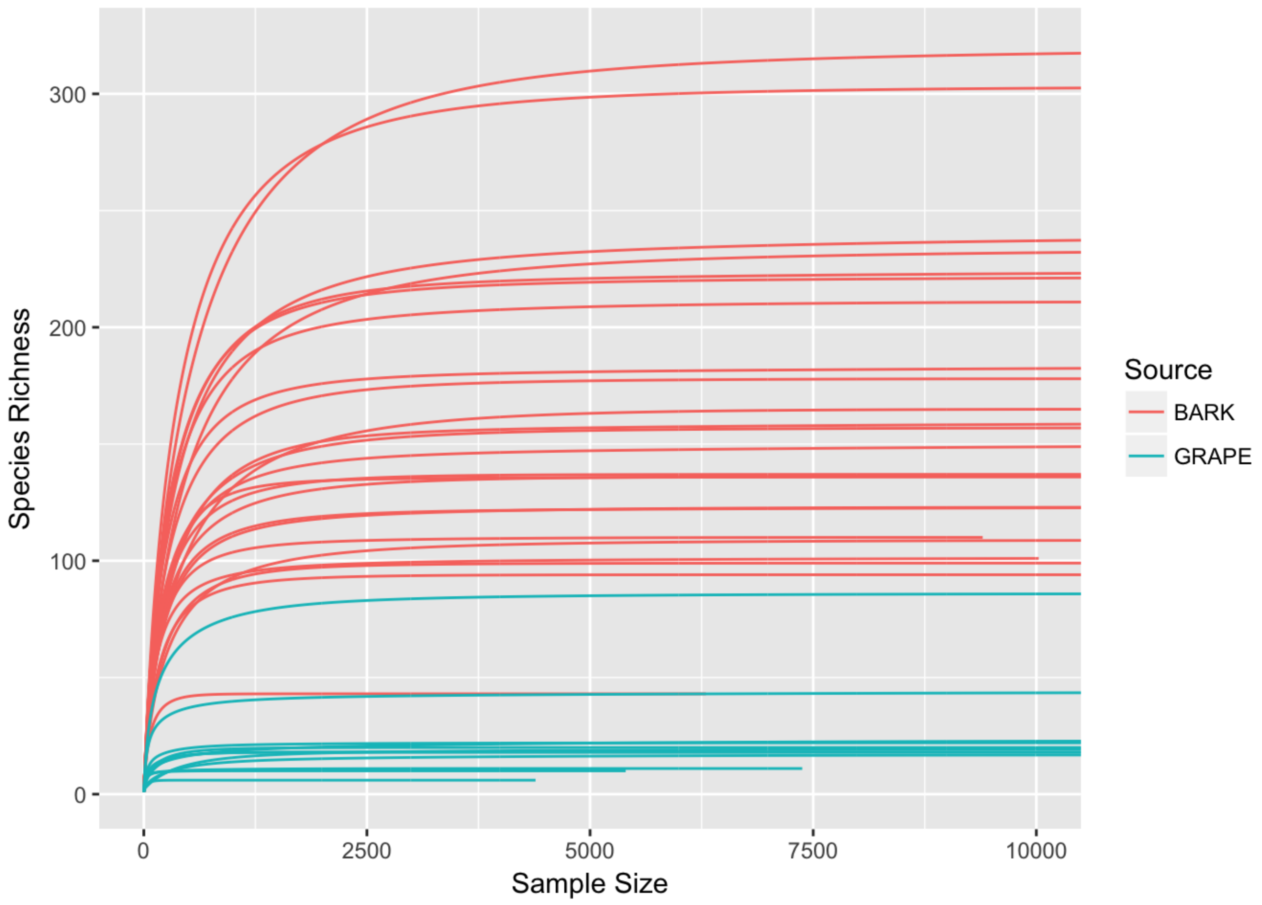

Supplementary Figure S2. Relative abundance of bacterial epiphytes in bark and grape samples

A. Barcharts at phylum level

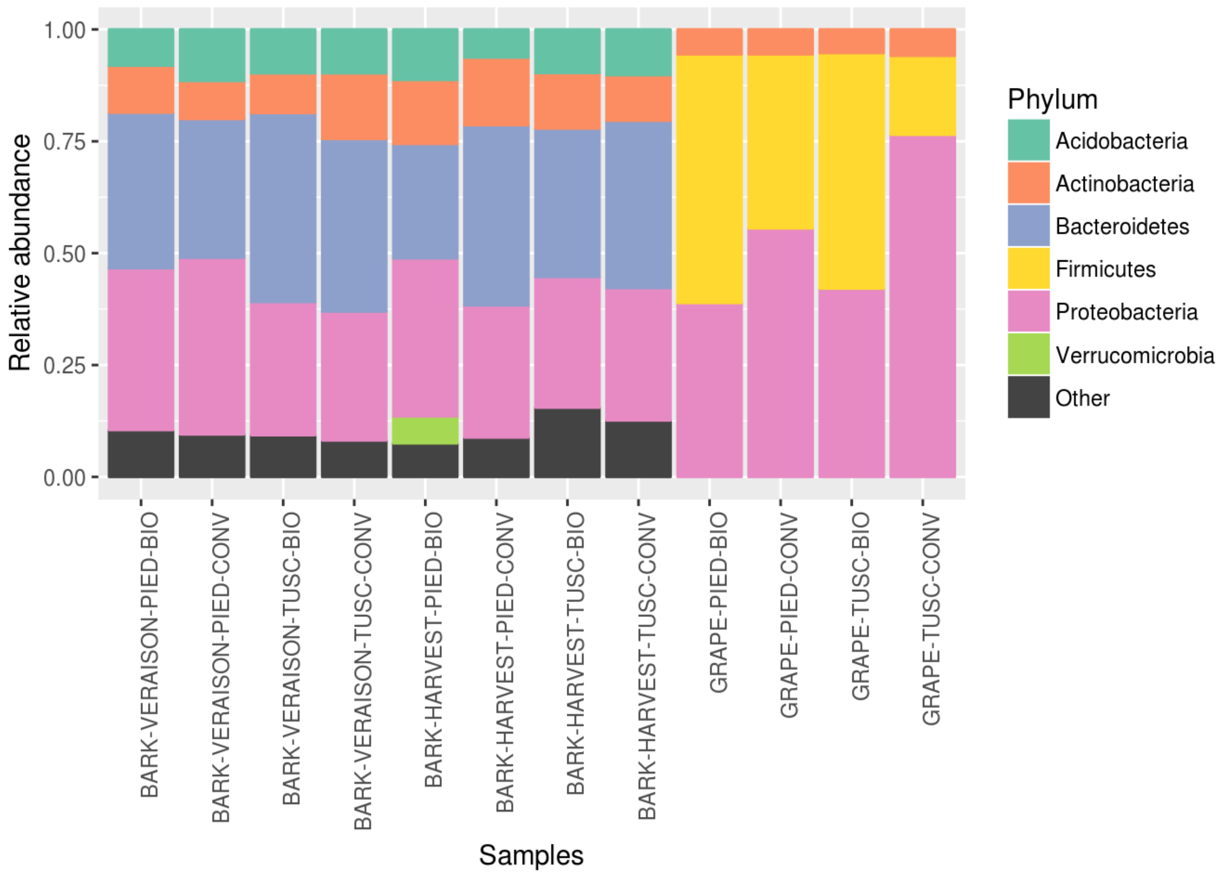

B. Barcharts at class level

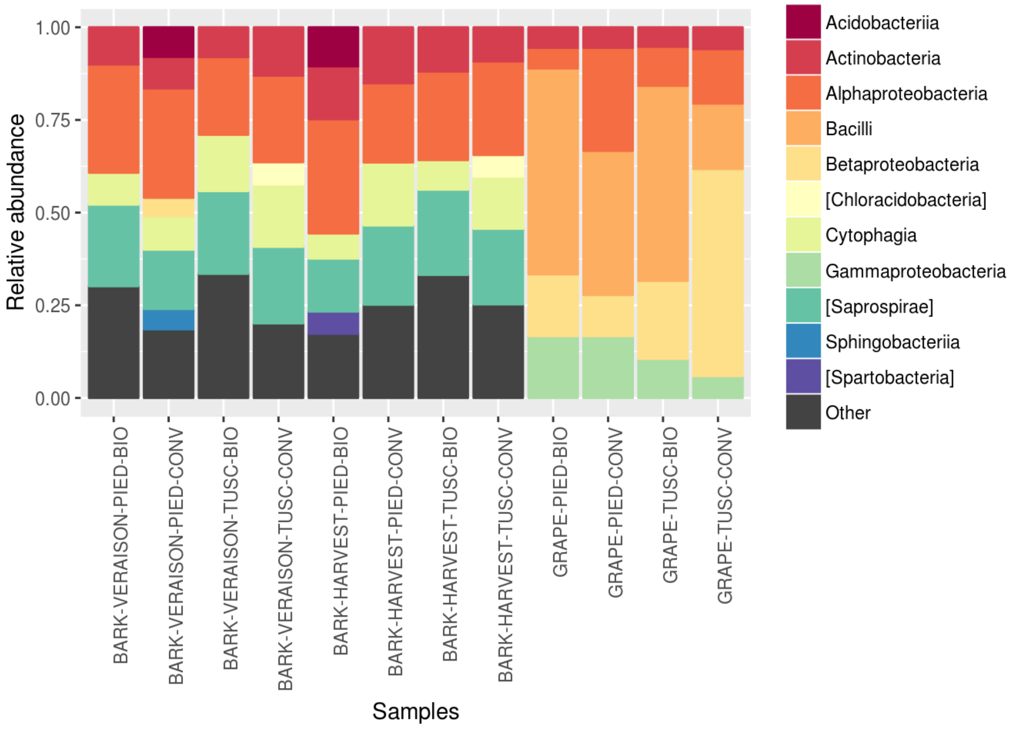

Supplementary Figure S3. sPLS-DA on BARK data-set

A. Score plot on components 1 and 2

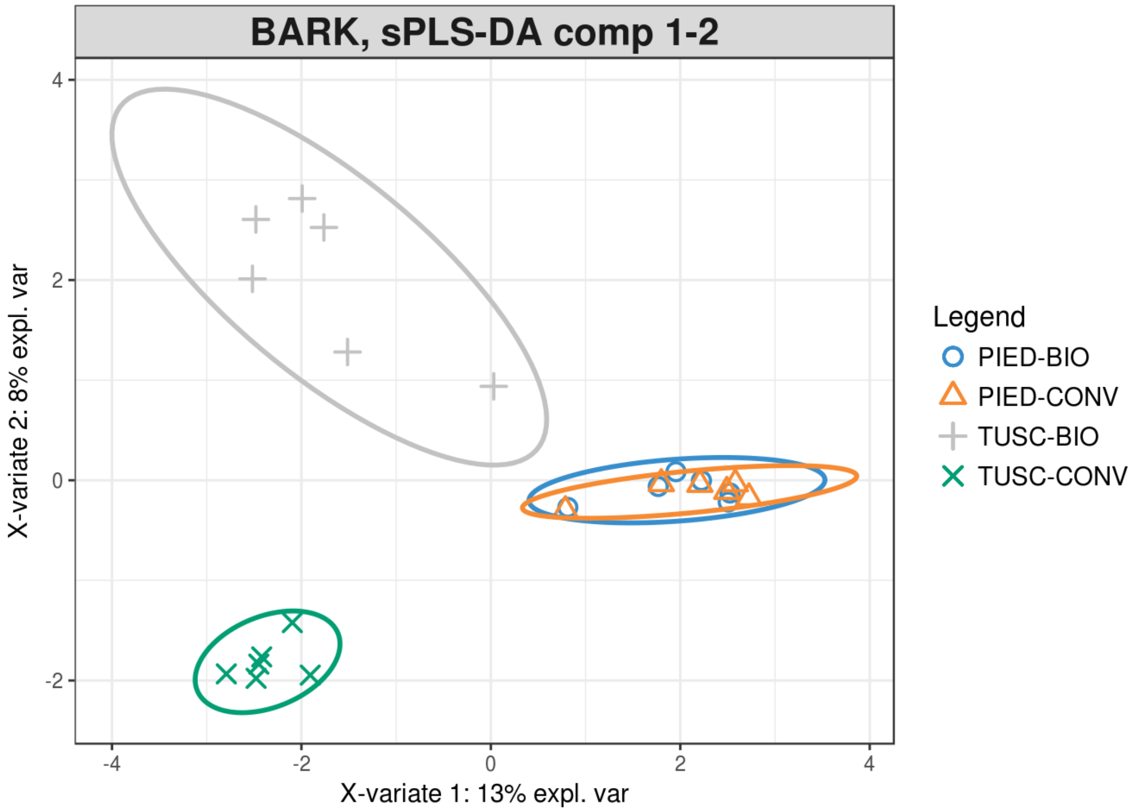

B. Score plot on components 2 and 3

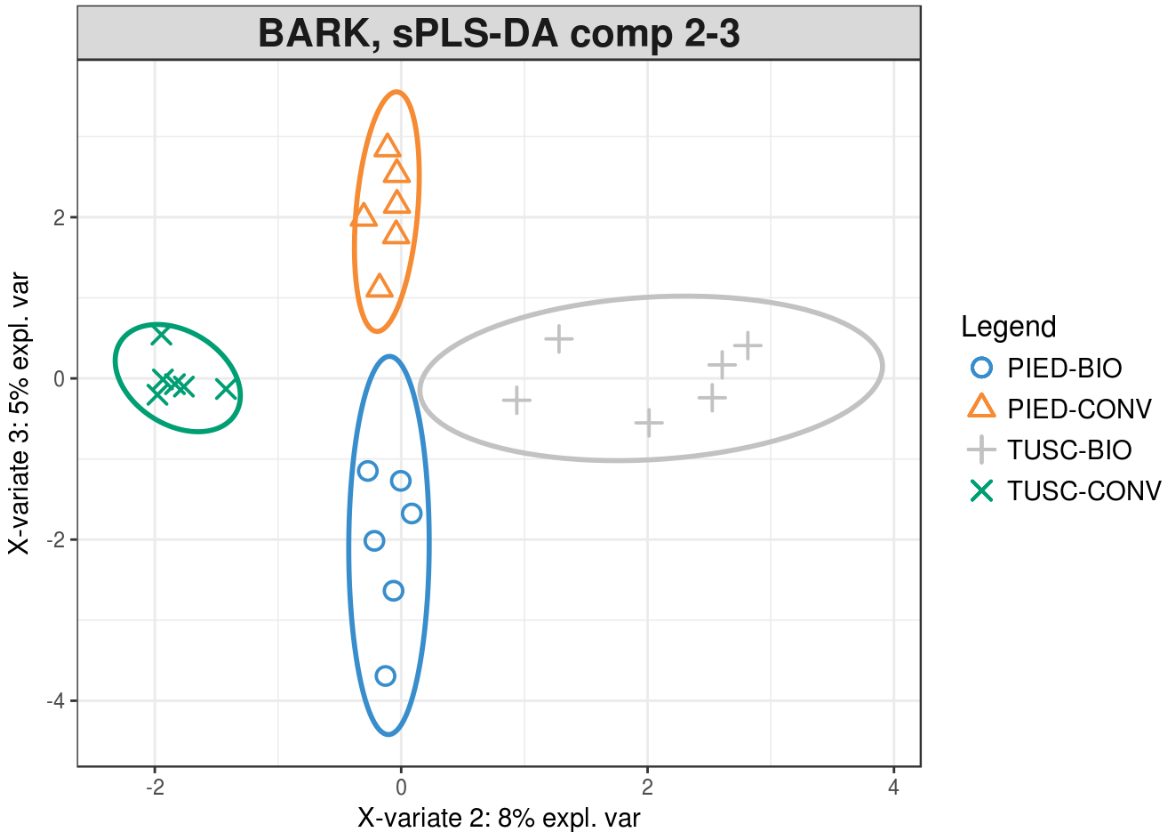

C. Single entries contribution on component 1

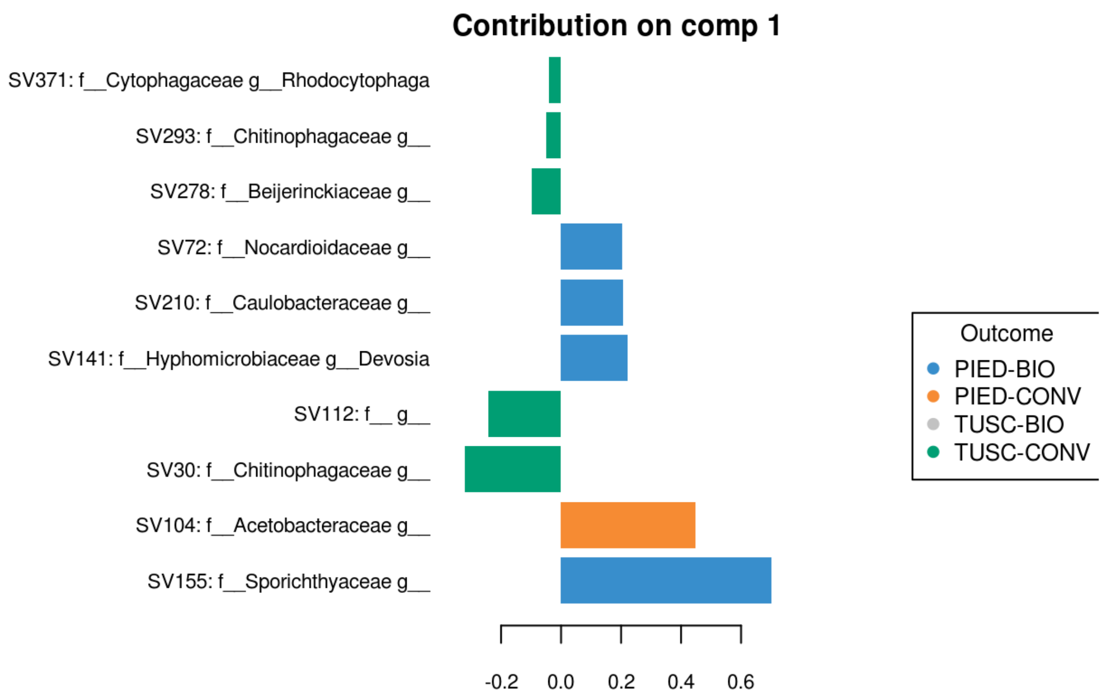

D. Single entries contribution on component 2

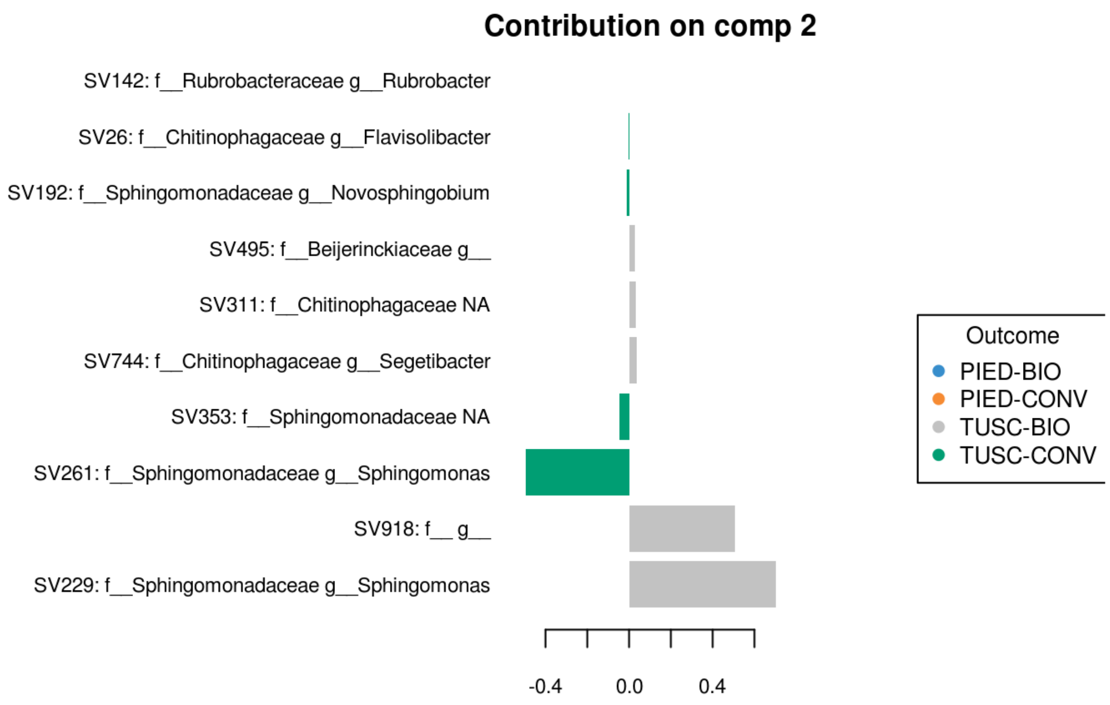

E. Single entries contribution on component 3

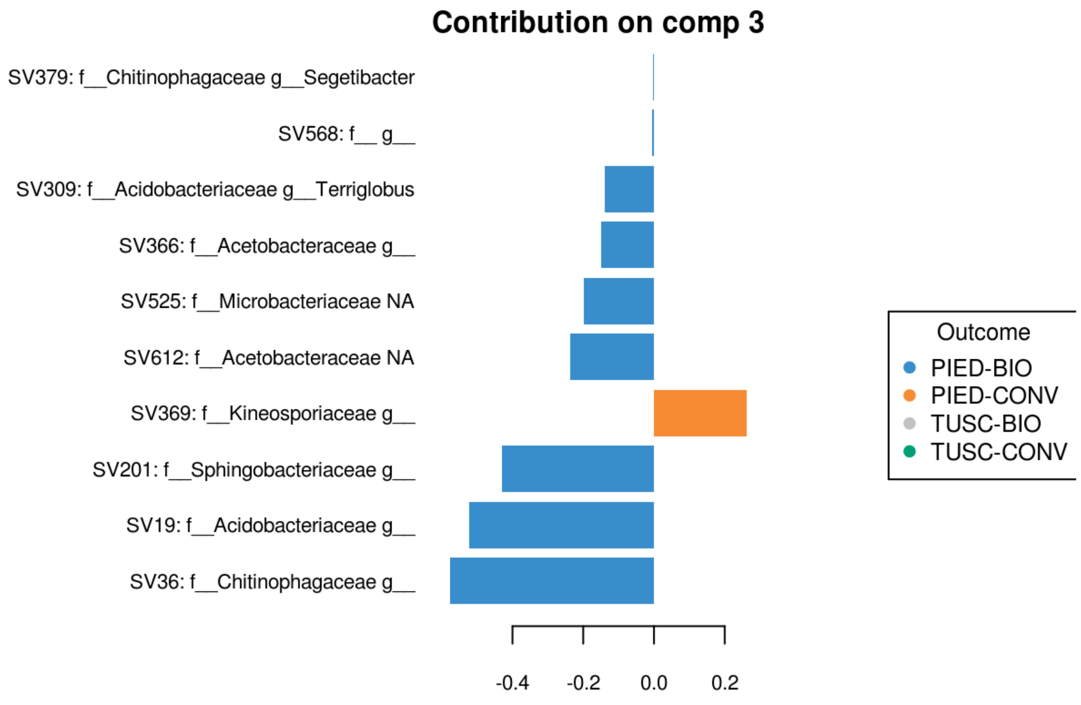

Supplementary Figure S4. sPLS-DA on grape data-set

A. Score plot on components 1 and 2

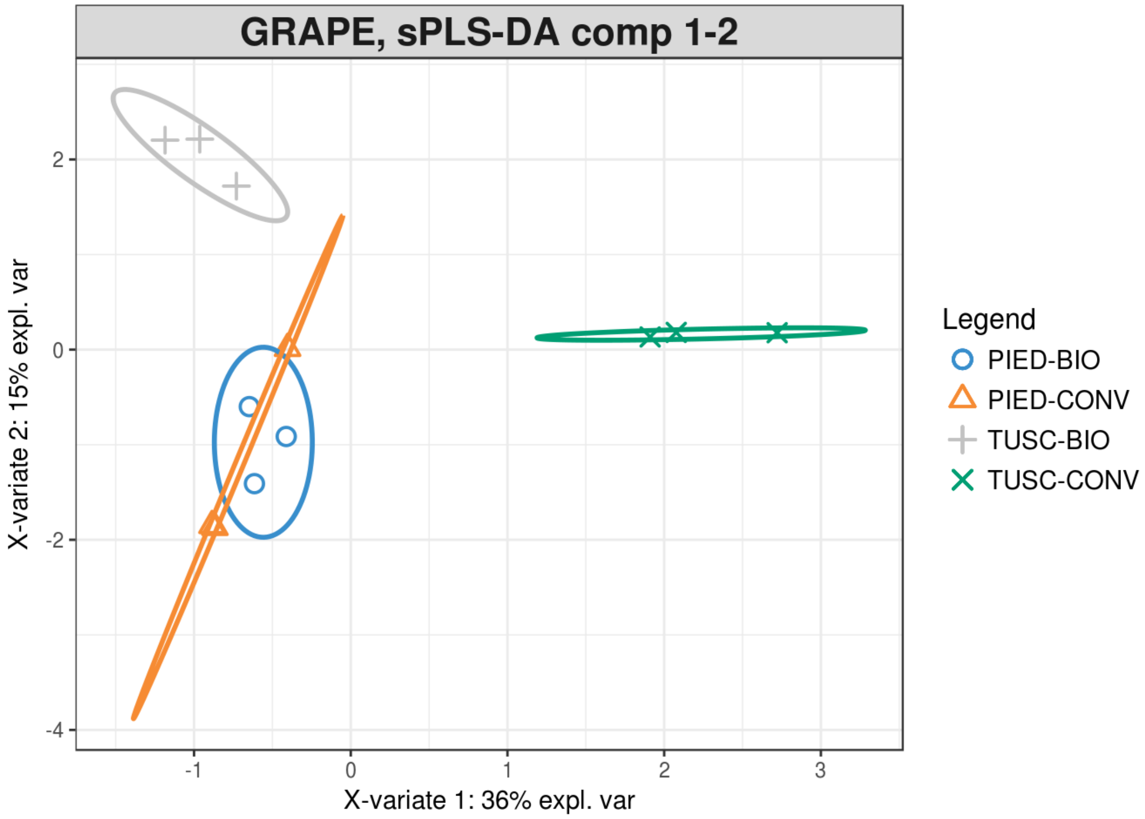

B. Score plot on components 2 and 3

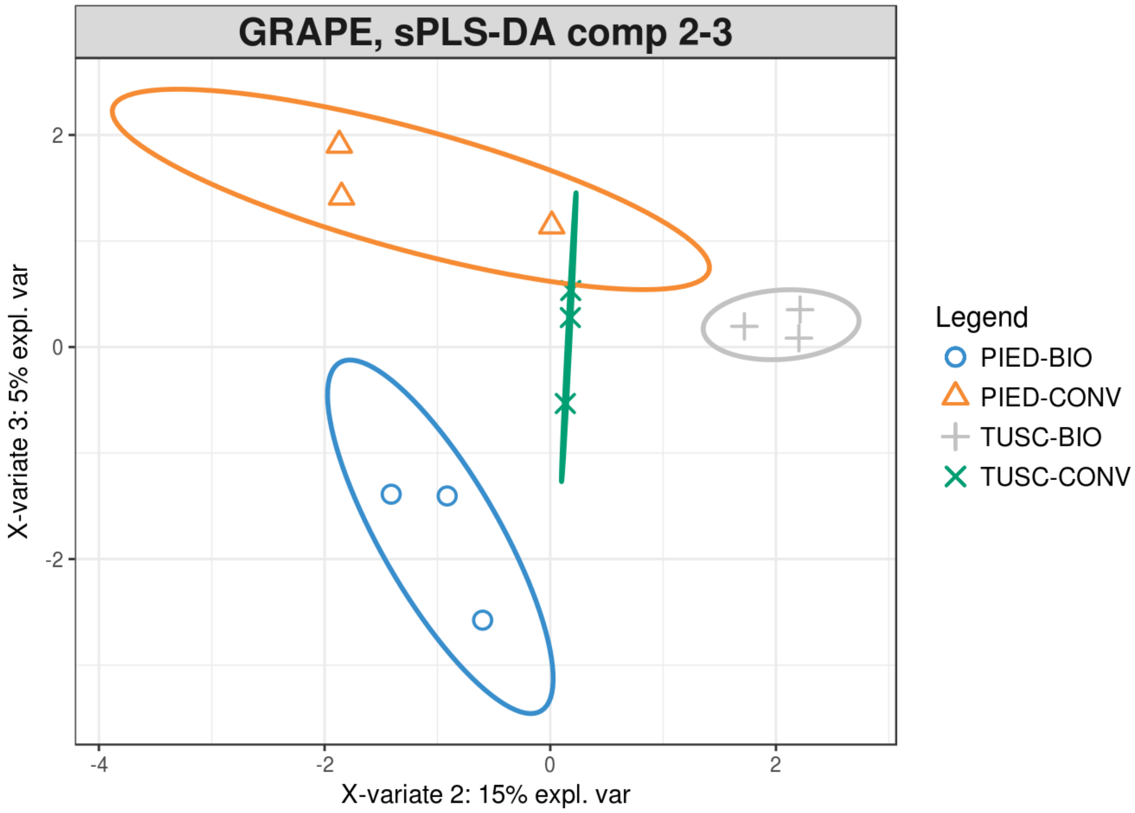

C. Single entries contribution on component 1

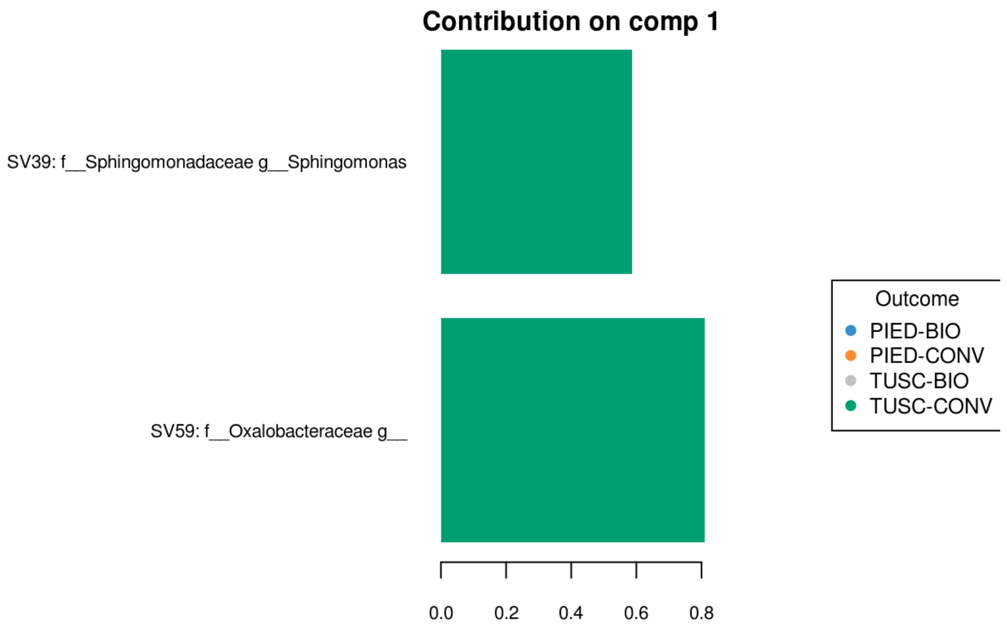

D. Single entries contribution on component 2

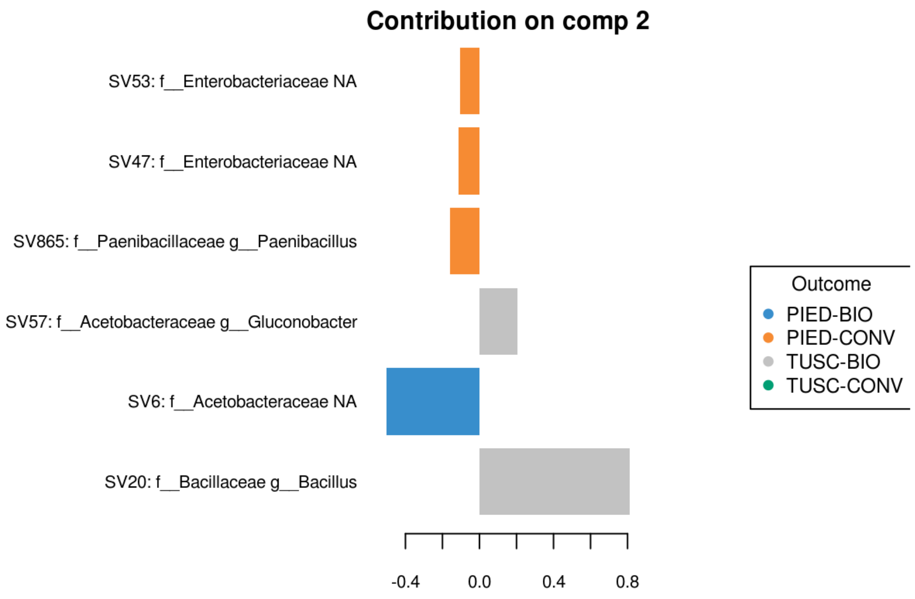

E. Single entries contribution on component 3

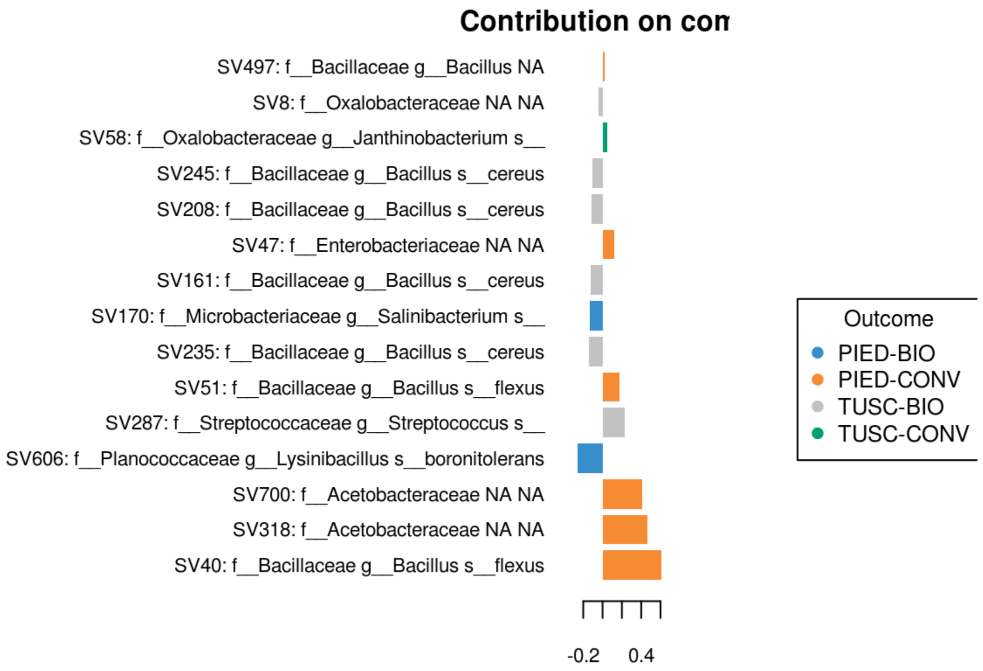

Supplement: Supplementary file 1 [file Data_Sheet_1.pdf]
